# Positive impacts of livestock and wild ungulate routes on functioning of dryland ecosystems

**DOI:** 10.1002/ece3.8147

**Published:** 2021-09-22

**Authors:** Ilan Stavi, Hezi Yizhaq, Yagil Osem, Eli Argaman

**Affiliations:** ^1^ Dead Sea and Arava Science Center Yotvata Israel; ^2^ Eilat Campus Ben‐Gurion University of the Negev Eilat Israel; ^3^ Department of Solar Energy and Environmental Physics Blaustein Institutes for Desert Research Ben‐Gurion University of the Negev Eilat Israel; ^4^ Department of Natural Resources Institute of Plant Sciences Volcani Center Rishon LeZion Israel; ^5^ Soil Erosion Research Station Ministry of Agriculture & Rural Development Bet Dagan Israel

**Keywords:** climate change, ecosystem engineering, herbivory effect, nontrophic effects, source–sink relations, water runoff

## Abstract

Livestock grazing is often perceived as being detrimental to the quality and functioning of dryland ecosystems. For example, a study in a semiarid Kenyan savanna proposed that cattle form bare spaces throughout the landscape, which indicate ecosystem degradation. Other studies, conducted in north‐eastern Spain, where climatic conditions range between semiarid and Mediterranean subhumid, reported that sheep and goat trails have increased the emergence of rill erosion processes. Sometimes, this negative perception is extended to include wild, large ungulate herbivores as well. Here, we challenge this perception by highlighting the generally nonadverse and even ameliorative impacts of moderate animal rate on geoecosystem functioning of hilly drylands. Specifically, trampling routes (also known as treading paths, livestock terracettes, cattle trails, migration tracks, cowtours, etc.) formed across hillslopes by grazing animals—being either domesticated livestock or native large herbivores—transform the original two‐phase vegetation mosaic of shrubby patches and interpatch spaces into a three‐phase mosaic. The animal routes increase the complexity of ecosystem, by strengthening the spatial redistribution of water and soil resources at the patch scale and decreasing hydrological connectivity at the hillslope scale. As a consequence, the animal routes improve functioning of hilly drylands and increase their resilience to long‐term droughts and climatic change. Therefore, instead of viewing the animal routes as degraded spots, they should be perceived at a wider perspective that allows to properly understand their overall role in sustaining dryland geoecosystems.

## Graphical abstract

Livestock animals and wild ungulates form trampling routes across the landscape, transforming the original two‐phase vegetation mosaic into a three‐phase mosaic. The routes increase the complexity of ecosystems and decrease hydrological connectivity at the hillslope scale, thus improving ecological functioning and sustaining dryland durability under long‐term droughts and climatic change.

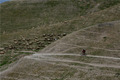

## 1 INTRODUCTION

In drylands, limited water availability cannot support full vegetation cover. Therefore, two‐phase mosaics, also known as source–sink ecosystems, are prevalent and form a patchy vegetation cover (Hoekstra & Shachak, 1999; Noy‐Meir, 1973), which can be highly heterogeneous. In relatively simple ecosystems, vegetation comprises mainly annual and perennial herbaceous species. In more complex systems, vegetation structure is made up of various life‐forms, including trees, shrubs, and herbaceous plants. Other plant forms, such as geophytes, as well as nonplant organisms, such as cyanobacteria, green algae, microfungi, moss, and lichen that comprise biological crusts, can also grow in these ecosystems. Depending on the complexity of the system, the vegetation patches form certain spatial patterns, such as stripes, strands, stipples, spots, and others (Ludwig et al., 1999; Meron, 2015). During rainstorms, some of the raindrops that fall upon the bare interpatch spaces infiltrate on‐site, while others flow overland as runoff and accumulate in the downslope vegetation patches, where soil conditions allow higher infiltrability (Stavi, Lavee, et al., 2009). The interpatch spaces form source areas for suspended and dissolved materials, which accumulate in the sink (vegetation) patches along with the runoff water (Pueyo et al., 2013). Evidence for two‐phase vegetation patterns has been reported for the entire range of drylands, spanning between dry subhumid regions at the moistest extreme and hyper‐arid regions at the driest extreme (Hoekstra & Shachak, 1999).

Recently, the term “geoecosystem functioning” has been introduced to demonstrate the complex relations and feedbacks between the physical and biotic components of natural or seminatural environments. Specifically, this term pertains to the capacity of land units to retain scarce resources of water and soil, and prevent their leakage outside of their boundaries (Stavi, Rachmilevitch, Hjazin, et al., 2018). In this regard, vegetation patches, and particularly shrub and tree plant species, fill an important role, as they considerably improve the soil quality and tremendously increase water infiltration capacity (Tongway & Ludwig, 2003). Over time, self‐organization of the vegetation cover regulates surface processes, decreasing hydrological connectivity and lowering soil erosion at the hillslope scale. Consequently, the patterned vegetation improves the resilience of ecosystems under long‐term droughts and climatic change (Meron, 2016).

## 2 IMPLICATIONS OF ANIMAL ROUTES FOR GEOECOSYSTEM FUNCTIONING

**FIGURE 1:**
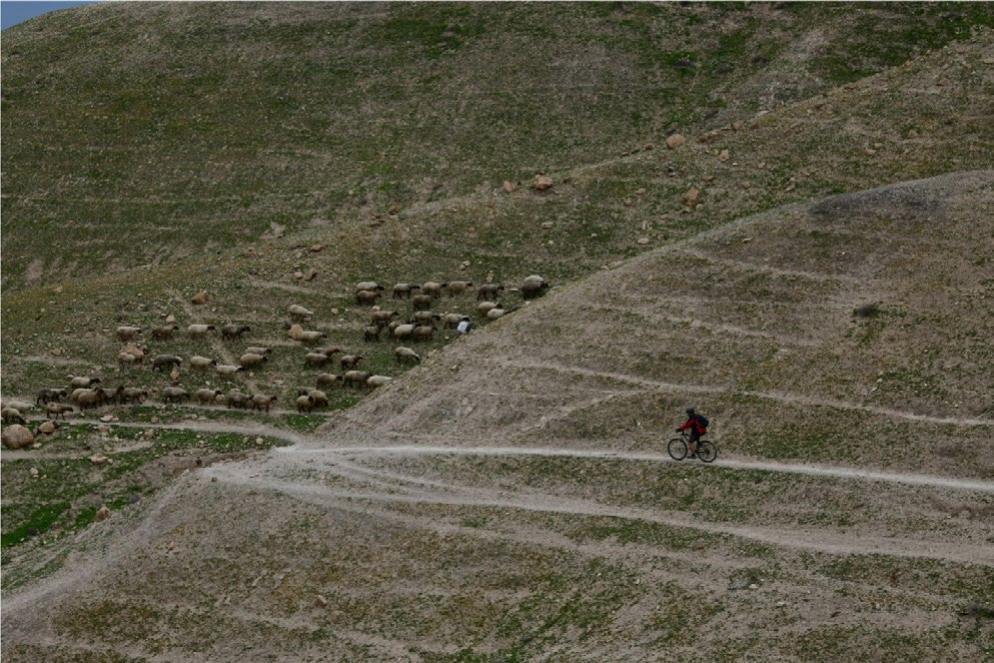
Livestock‐made trampling routes. Note the characteristic movement of the sheep flock in parallel lines across the hillslope. The picture was taken by H. Yizhaq in the Israeli hyper‐arid southern Judean Desert, which is prone to long‐term grazing by sheep and goats

Grazing ungulates, being either domesticated livestock or wild large herbivores, substantially impact geoecosystem functioning (Stavi et al., 2012; Stavi, Shem‐Tov, et al., 2015). In addition to the direct and indirect effects of plant material consumption, the animals also impact the system through nontrophic effects (Stavi, Lavee, et al., 2009; Stavi, Rachmilevitch, & Yizhaq, 2018). One of the most prominent nontrophic impacts is caused by the trampling or treading action of the ungulate, also named hoof action or hoof mechanism (Stavi et al., 2008; Stavi, Unger, et al., 2009). Essentially, the animals' hooves tear herbaceous vegetation, shear the ground surface, and compact the soil (Bilotta et al., 2007). However, the impact exerted by animals is not evenly distributed in space. This particularly pertains to mountainous, hilly, or undulating landscapes, where animal movements are determined by the landform settings. Specifically, herds or flocks of ungulates characteristically tread in parallel lines, roughly along contours (Figure 1; see also Figure 2 in Rahmanian et al., 2019). This mode of movement stems from the animals' desire to save energy while moving from one site to another (Coughenour, 1991; Ganskopp et al., 2000).

**FIGURE 2:**
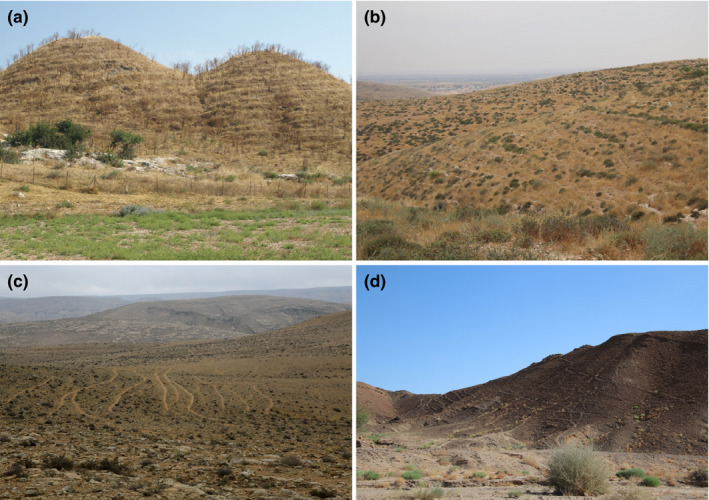
Ungulate‐made trampling routes transecting hillslopes. Notes: A dry subhumid rangeland prone to long‐term grazing by domesticated cattle. The picture was taken by I. Stavi in the Ramot Menashe region of northern Israel (a); a semiarid land prone to long‐term grazing by domesticated sheep and goats. The picture was taken by I. Stavi in the northern Negev of Israel (b); an arid region prone to long‐term grazing by domesticated sheep and goats. The picture was taken by H. Yizhaq in the central Negev of Israel (c); a hyper‐arid nature reserve, where ungulate population is dominated by the (reintroduced) native Asiatic wild ass (*Equus hemionus*). The picture was taken by I. Stavi in the Makhtesh Ramon region of southern Israel (d)

In the hilly semiarid northern Negev region of Israel, it has been reported that the repetitive movement of flocks of sheep and goats along certain paths turns them into definite routes, which are distinctly different from the interpatch spaces (Stavi et al., 2008, 2012; Stavi, Rachmilevitch, & Yizhaq, 2018; Stavi, Shem‐Tov, et al., 2015; Stavi, Ungar, et al., 2009). Across these hilly rangelands, trampling routes cover approximately 10% of the hillslopes' ground surface (Stavi, Shem‐Tov, et al., 2015). Similar routes are formed either by domesticated livestock or by wild large herbivores, and are common in a wide range of climatic regions. For example, in Israel, animal routes occur between the dry subhumid region in the north of the country, through the central, semiarid, and arid zones, to the hyper‐arid region in the south of the country (Figure 2).

Similar route patterns have been reported for other drylands around the world, such as in China (Jin et al., 2016, 2019; Sun et al., 2020), Kyrgyzstan (Liu & Watanabe, 2013), Spain (Ries, 2010; Ries et al., 2013), and the USA (Corrao et al., 2015, 2016; Ganskopp et al., 2000; Trimble & Mendel, 1995). Also, despite not specifically indicated, livestock routes have apparently formed in Iran (see Rahmanian et al., 2019). In different parts of the world, ungulate routes have received different names, including trampling tracks, treading paths, animal terraces, migration trails, livestock terracettes, cowtours, all of which represent similar patterns.

The unique properties of ungulate routes define them as a distinct microhabitat. Above all, the routes are characterized by comparatively high soil compaction, which results in high surface penetration resistance and soil bulk density (Stavi et al., 2008; Stavi, Ungar, et al., 2009). This effect was demonstrated in hilly rangelands of the semiarid Negev by comparing the ground surface penetration resistance of active routes with that of nonactive routes (in decade‐old exclosures). While in active routes, the average penetration resistance was as high as 0.44 MPa, in the nonactive routes, it was only 0.17 MPa (Stavi, Ungar, et al., 2009: averages are modified from penetration depth, in mm).

**FIGURE 3:**
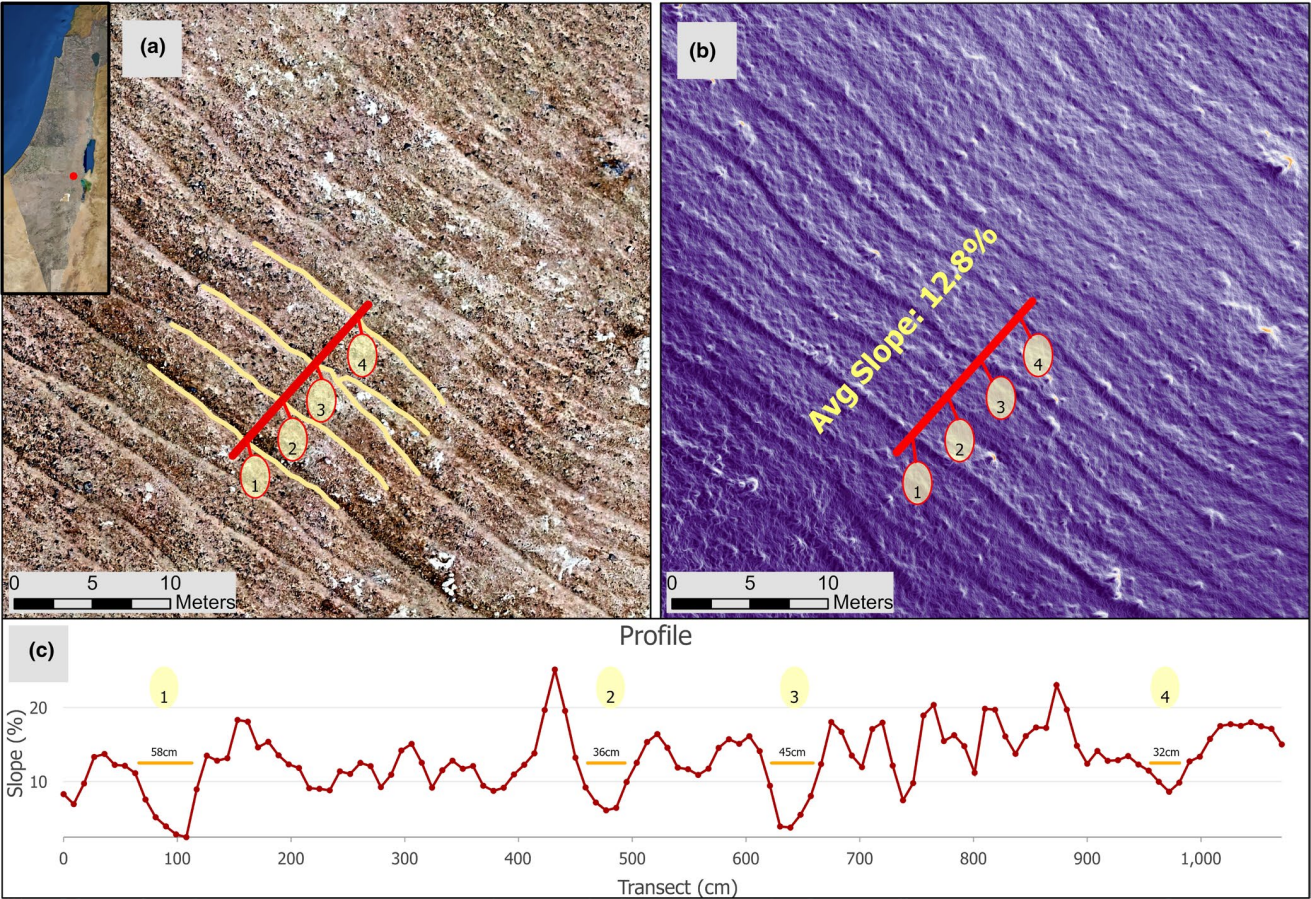
Fine‐resolution orthophotograph of a representative hillslope (31°16′50.5″N, 35°14′13.9″E) in the hyper‐arid south‐eastern Negev of Israel. Notes: A 1.5 cm/px orthophotograph (panel a). A high‐resolution calculation of a characterizing hillslope, demonstrating the downslope descent rate of change in the trampling routes along the sampled transect (panel b). Width is presented for the routes (orange lines), and incline is presented for the entire transect (panel c). Ground surface mapping was conducted using an unmanned aerial vehicle (UAV), acquired in August 2020, using a MAVIC PRO quadcopter equipped with a 12.35 MP RGB camera (DJI Technology Co., Shenzhen, China) operated by DJI Ground Station Pro®. Flight altitude was 50 m above ground level, with an overlap of 85% for photogrammetric processing done with Agisoft Metashape Pro® (version 1.6.3, https://www.agisoft.com/). Postprocessing of the UAV images resulted in orthomosaics with 1.5 cm/px spatial resolution and a 3.0 cm/px digital elevation model (DEM). The incline (%) was calculated using the SLOPE algorithm of ArcGIS Pro (ESRI ver. 2.7.0) run over the DEM raster. The average hillslope incline is 12.8%, while the mean catenary incline of the trampling routes is 3.3%

It has been proposed that trampling routes increase the complexity of dryland ecosystems, turning the original two‐phase mosaics into three‐phase mosaics (Stavi et al., 2012). Specifically, because of the low water infiltrability of the routes' ground surface, it has been suggested that the routes modify the spatial redistribution of water and suspended/dissolved resources at the patch scale. Studies have shown that the routes are “net contributors” (net source areas) of runoff and associated resources, which accumulate in the downslope vegetation patches (Stavi, Rachmilevitch, & Yizhaq, 2018; Stavi, Shem‐Tov, et al., 2015).    Additionally, it was shown that the routes modify the comparatively homogeneous profile of hillslopes, intensifying its step‐like structure. This effect was demonstrated for the semiarid hilly northern Negev by measuring the ground surface incline along catenary transects. The average catenary incline of active routes was 5.7°, whereas the catenary incline of routes in decade‐old exclosures was 10.2° (Stavi, Ungar, et al., 2009). This effect is further substantiated by 3D modeling of a representative hillslope in the hyper‐arid north‐eastern Negev, which is transected by animal routes. The model shows that the average catenary incline of the routes is 3.3% (2.0°), while the general incline of the hillslopes is 12.8% (7.3°) (Figure 3).

**FIGURE 4:**
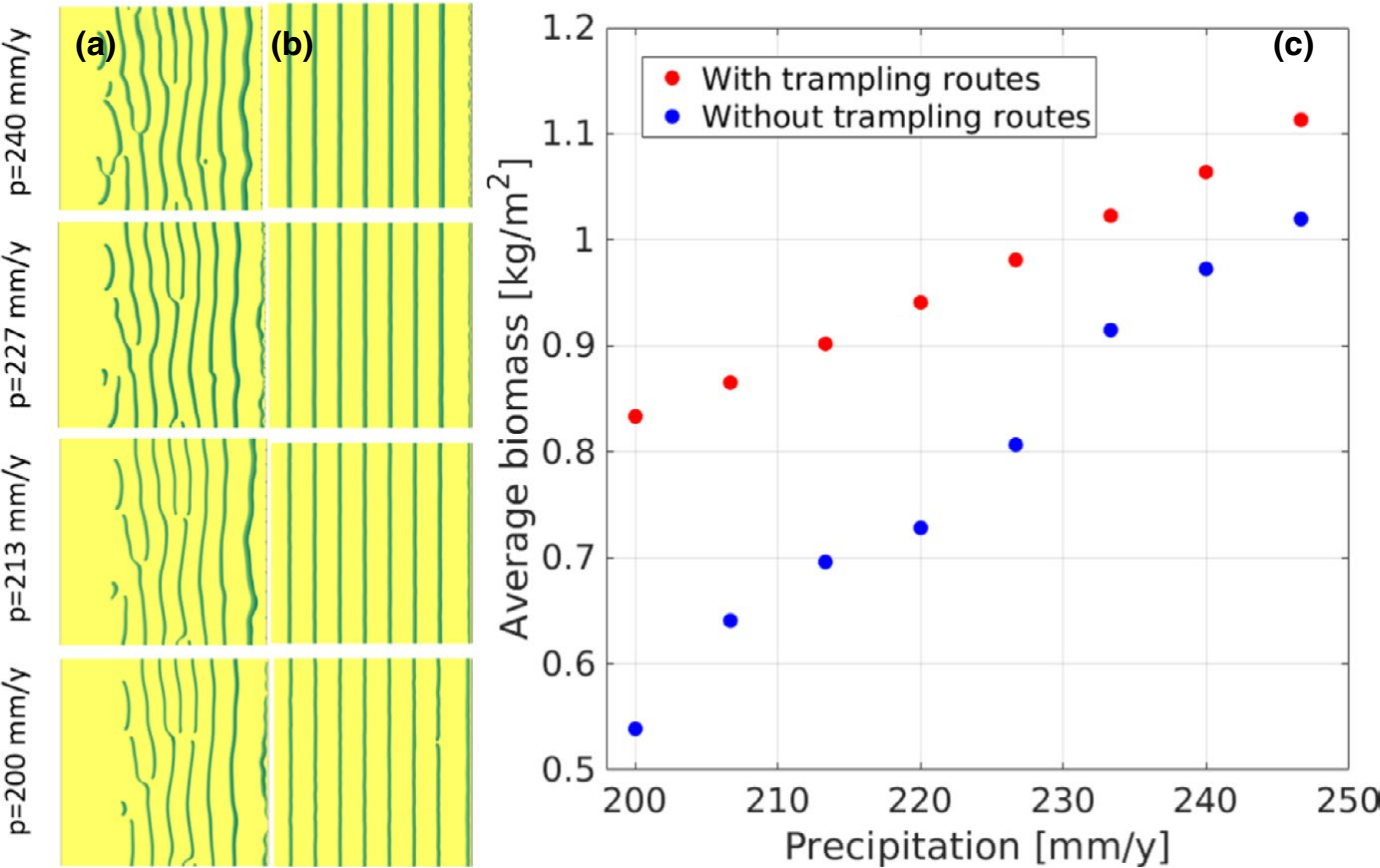
Mathematical modeling of the impact of trampling routes on vegetation pattern and biomass under decreasing precipitation regime. Notes: The simulations are for 5° hillslopes. The vegetation pattern (stripes or banded vegetation perpendicular to the slope that developed spontaneously from random initial conditions) is shown for hillslopes without trampling routes (column a), and for hillslopes with 8 trampling routes defined by lower infiltration values (column b), under 4 annual precipitation rates (the green lines indicate vegetation, and the yellow background indicates bare soil). The average biomass density along the precipitation gradient is shown in panel c. The average biomass in hillslopes with trampling routes is considerably greater than that in hillslopes without trampling routes throughout the modeled precipitation range. For more details on the mathematical model, see Appendix S1

It has been suggested that the compacted, smooth surface of the routes increases hydrological connectivity at the patch scale, increasing the contribution of runoff water to the downslope vegetation patches. At the same time, the intensified step‐like profile of the ground surface lessens hydrological connectivity at the hillslope scale, lowering the leakage of runoff water out of the system (Stavi, Rachmilevitch, & Yizhaq, 2018). Therefore, the overall nontrophic impact of animal routes on the functioning of dryland geoecosystems is not adverse. Moreover, it improves the hydrological functioning of ecosystems by increasing their resilience to long‐term droughts and climate change (Stavi, Lavee, et al., 2009; Stavi, Rachmilevitch, & Yizhaq, 2018; Stavi, Shem‐Tov, et al., 2015). This effect was verified by mathematically modeling the impact of animal routes on shrubby vegetation under decreasing precipitation regimes. The model shows that the presence of routes mitigates the decrease in vegetation biomass under long‐term drought conditions. Specifically, the model demonstrates that under “normal” precipitation regime, the ecosystem's net primary productivity (NPP) is ~10% greater in ecosystems with animal routes than that in ecosystems without routes, while under drought conditions, the difference between them can increase to ~50% (Figure 4). Thus, the modification of landforms by ungulate routes can be considered ecosystem engineering, in which key species of animals, plants, or microorganisms regulate—through nontrophic effects—the productivity of other organisms by controlling their access to resources or by modifying their habitat conditions (see Gilad et al., 2004; Jones et al., 1994, 1997).

The trampling routes not only redistribute water and soil resources at the patch and hillslope scales but they also regulate the vegetation structure through zoochory, including both endo‐ and epi‐zoochory mechanisms (Aschero & García, 2012). For example, both livestock animals and wild ungulates serve as effective vectors of plant seeds, which pass through their gastrointestinal system (Faust et al., 2011; Stavi, Zinnes, et al., 2015). The excreted dung along the routes (Lange, 1969) affects seed dispersal and germination, and modifies vegetation composition. Also, seeds and pollen may be transported by the animals' fur and hooves (Kaligarič et al., 2016) and then redeposited along the routes. Further, seeds buried by the hoof action receive better conditions for germination and growth (Eichberg & Donath, 2018; Faust et al., 2011).

## 3 POSSIBLE LIMITATIONS

The positive effects of routes on geoecosystem functioning seem to be valid as long as animal rate (number of animals per unit of land per unit of time: Coughenour, 1991) is moderate (Stavi, Rachmilevitch, & Yizhaq, 2018; Stavi, Shem‐Tov, et al., 2015). At the same time, high animal rate can be detrimental to the functioning of the land unit. A possible adverse impact is the substantial decrease in herbaceous vegetation cover, with the consequent excess expansion of bare patches. For example, in a semiarid Kenyan savanna, cattle grazing was reported to form degraded bare spaces throughout the landscape. Yet, long‐term grazing exclusion has led to revegetation of the bare spaces by herbaceous plants, demonstrating the potential reversal of degradation processes and recovery of these rangelands (Augustine et al., 2019). Further, overgrazing may entirely remove vegetation, consequently simplifying the ground surface and preventing ecosystem self‐organization. This effect is expected to increase hydrological connectivity at the hillslope scale, accelerating erosional processes and causing land degradation (Gamoun et al., 2010; Stavi, Shem‐Tov, et al., 2015).

**FIGURE 5:**
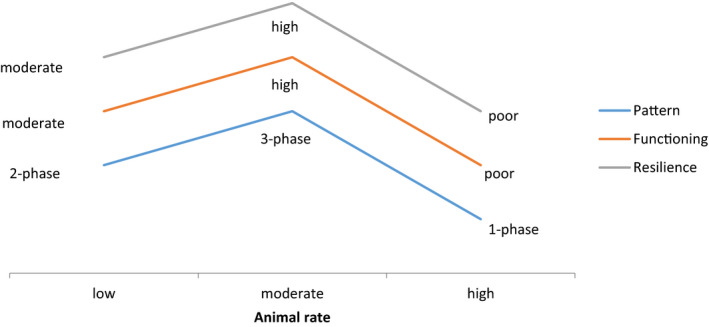
Animal rate impact on vegetation pattern, ecosystem functioning, and resilience under long‐term droughts and climatic change. Note: Modified from Stavi, Shem‐Tov, et al. (2015)

An alternative adverse impact is the excess increase in spatial redistribution of water as overland flow, which accumulates in the woody vegetation patches that expand at the expense of herbaceous (forage) vegetation. Despite the expected increase in the ecosystem's NPP, this chain of effects decreases ecosystem complexity, reduces plant species diversity, and lowers the economic value of rangelands (Schlesinger et al., 1990; Stavi, Shem‐Tov, et al., 2015). At the same time, if animal rate is too low, or where animal grazing is excluded, herbaceous (Augustine et al., 2019) or woody vegetation cover may increase, decreasing or eliminating the bare interpatch spaces (Archer et al., 2017; Stavi, Lavee, et al., 2009; Turner et al., 2003). A similar effect was reported for the northern Negev region, where goats grazing on the fresh foliage of the dominant shrub species, *Sarcopoterium spinosum* (L.) Spach, regulate its cover (Stavi et al., 2008). Whether the vegetation is herbaceous or woody, increased plant cover decreases the runoff source areas of bare spaces, lessening spatial redistribution of water (Durán Zuazo & Rodríguez Pleguezuelo, 2008; Stavi, Rachmilevitch, Hjazin, et al., 2018) and lowering geoecosystem resilience under potentially degraded climatic conditions (Stavi, Rachmilevitch, Hjazin, et al., 2018). The impact of animal rate on the ecosystem's vegetation pattern, hydrological functioning, and durability under long‐term droughts and climatic change is schematically illustrated in Figure 5.

As described above, this work emphasizes the mechanism through which animal routes impact the functioning of mountainous, hilly, or undulating land units. In such landscapes, the inclined surface is a precondition for the formation of these routes. To some extent, this is consistent with Sun et al. (2020), who reported that in the Chinese Loess Plateau, sheep terracettes occur only in steep hillslopes (incline >30°) and are generally absent in comparatively gentle hillslopes (incline <30°). However, in Oregon, the USA, cattle routes were observed in moderate hillslopes, with an average incline of ~4.5° (Ganskopp et al., 2000). Although their impact on geoecosystem functioning is generally positive, it seems that under certain circumstances, animal routes may accelerate soil erosional processes. For example, across the semiarid and subhumid regions of Spain, sheep and goat routes were reported to accelerate rill erosion (Ries, 2010; Ries et al., 2013). Specifically, average runoff coefficient and sediment discharge from route surface were ~30% and almost fivefold greater, respectively, than in nonroute surface (Ries, 2010). The tremendous increase in sediment yield from route surfaces was attributed to the hoof action that shears and loosens mineral material, which then becomes available for transport (Ries et al., 2013).

Regardless, the formation of animal routes has also been reported in flat land units and plains, where their occurrence was proposed to affect the emergence and establishment of seedlings, as well as the structure and composition of vegetation (Eichberg et al., 2008; Rosenthal et al., 2012). Particularly, this topic has been extensively studied with respect to the ecological impacts of livestock on the surroundings of water points (piosphere). For example, the density of livestock routes (Lange, 1969) and number of dung pellets (Lange, 1969; Walker & Hkitschmidt, 1986) were reported to increase with proximity to water points. Despite not being specifically assessed, it was suggested that livestock routes increase soil erosion in piosphere environments (Walker & Hkitschmidt, 1986). One way or another, it is known that treading routes can also be formed by nonungulate (nonhoofed) animals, such as African elephants (*Loxodonta africana*: Dai et al., 2007), gopher tortoises (*Gopherus polyphemus*: Halstead et al., 2007), and others.

## 4 RESEARCH GAPS

Additional studies are required to answer open questions regarding the mechanisms through which animal routes affect the geoecosystem functioning of drylands. For example: (1) How do lithology, topography, and soil type affect route formation and pattern?; (2) How are the morphology and patterns of routes regulated by regional climatic conditions (in the long term), and by local climatic fluctuations (in the short term)?; (3) What is the optimal cover of animal routes to maximize forage production while sustaining geoecosystem functioning?; (4) What is the best animal rate to form the optimal cover of routes?; (5) How does the routes' optimal cover change for hillslopes with different inclines or of different shapes (concave vs. convex morphology)?; (6) How does the routes' optimal cover change for hillslopes with different aspects (e.g., north‐ vs. south‐facing hillslopes)?; (7) How does the routes' optimal cover change across climatic gradients?; and (8) How is the impact of animal routes on the spatial distribution of soil‐water at the patch and hillslope scales regulated by the abovementioned issues?

These and other questions necessitate thorough research of this topic, which has direct implications for elemental cycling, ecosystem health, and environmental sustainability. Global climatic change, with the forecasted aggravation of aridity in the world's deserts and the expansion of dryland areas (see Cook et al., 2014; Fu & Feng, 2014; Lickley & Solomon, 2018), emphasizes the importance and applicability of such future studies.

## CONFLICT OF INTERESTS

The authors declare no conflict of interests.

## AUTHOR CONTRIBUTIONS

**Ilan Stavi:** Conceptualization (lead); project administration (lead); resources (lead); supervision (lead); writing‐original draft (lead). **Hezi Yizhaq:** Conceptualization (supporting); software (equal); writing‐original draft (equal); writing‐review & editing (equal). **Yagil Osem:** Conceptualization (equal); investigation (equal); validation (equal); writing‐review & editing (equal). **Eli Argaman:** Conceptualization (equal); investigation (equal); writing‐original draft (equal); writing‐review & editing (equal).

## Supporting information

Appendix S1

## Data Availability

The paper is a Viewpoint, with no additional data.

## References

[ece38147-bib-0001] Archer, S. R. , Andersen, E. M. , Predick, K. I. , Schwinning, S. , Steidl, R. J. , & Woods, S. R. (2017). Woody plant encroachment: Causes and consequences. In D. D. Briske (Ed.), Rangeland systems – Processes, management and challenges (pp. 25–84). Springer.

[ece38147-bib-0002] Aschero, V. , & García, D. (2012). The fencing paradigm in woodland conservation: Consequences for recruitment of a semi‐arid tree. Applied Vegetation Science, 15, 307–317. 10.1111/j.1654-109X.2011.01180.x

[ece38147-bib-0003] Augustine, D. J. , Wigley, B. J. , Ratnam, J. , Kibet, S. , Nyangito, M. , & Sankaran, M. (2019). Large herbivores maintain a two‐phase herbaceous vegetation mosaic in a semi‐arid savanna. Ecology and Evolution, 9, 12779–12788. 10.1002/ece3.5750 31788213 PMC6875565

[ece38147-bib-0004] Bilotta, G. S. , Brazier, R. E. , & Haygarth, P. M. (2007). The impacts of grazing animals on the quality of soils, vegetation, and surface waters in intensively managed grasslands. Advances in Agronomy, 94, 237–280.

[ece38147-bib-0005] Cook, B. I. , Smerdon, J. E. , Seager, R. , & Coats, S. (2014). Global warming and 21st century drying. Climate Dynamics, 43, 2607–2627. 10.1007/s00382-014-2075-y

[ece38147-bib-0006] Corrao, M. V. , Cosens, B. E. , Heinse, R. , Eitel, J. U. H. , & Link, T. E. (2015). Using science to bridge management and policy: Terracette hydrologic function and water quality best management practices in Idaho. Rangelands, 37, 191–199. 10.1016/j.rala.2015.08.003

[ece38147-bib-0007] Corrao, M. , Heinse, R. , Eitel, J. , Cosens, B. , & Link, T. (2016). Soil moisture differences between terracette benches and risers on semiarid rangeland hillslopes. Vadose Zone Journal, 15, vzj2015. 10.2136/vzj2015.04.0058

[ece38147-bib-0008] Coughenour, M. B. (1991). Spatial components of plant‐herbivore interaction in pastoral, ranching, and native ungulate ecosystems. Journal of Range Management, 44, 530–542.

[ece38147-bib-0009] Dai, X. , Shannon, G. , Slotow, R. , Page, B. , & Duffy, K. J. (2007). Short‐duration daytime movements of a cow herd of African elephants. Journal of Mammalogy, 88, 151–157. 10.1644/06-MAMM-A-035R1.1

[ece38147-bib-0010] Durán Zuazo, V. H. , & Rodríguez Pleguezuelo, C. R. (2008). Soil‐erosion and runoff prevention by plant covers. A review. Agronomy for Sustainable Development, 28, 65–86. 10.1051/agro:2007062

[ece38147-bib-0011] Eichberg, C. , Boes, J. , & Schwabe, A. (2008). Which vegetation and seed‐bank changes are induced by the disturbance regime of livestock trails in open sand ecosystems? Abhandlungen Aus Dem Westfälischen Museum Für Naturkunde, 70, 63–80.

[ece38147-bib-0012] Eichberg, C. , & Donath, T. W. (2018). Sheep trampling on surface‐lying seeds improves seedling recruitment in open sand ecosystems. Restoration Ecology, 26, S211–S219.

[ece38147-bib-0013] Faust, C. , Eichberg, C. , Storm, C. , & Schwabe, A. (2011). Post‐dispersal impact on seed fate by livestock trampling – A gap of knowledge. Basic and Applied Ecology, 12, 215–226. 10.1016/j.baae.2011.02.009

[ece38147-bib-0014] Fu, Q. , & Feng, S. (2014). Responses of terrestrial aridity to global warming. Journal of Geophysical Research: Atmosphere, 119, 2014JD021608.

[ece38147-bib-0015] Gamoun, M. , Tarhouni, M. , Belegacem, A. O. , Hanchi, B. , & Neffati, M. (2010). Effect of grazing and trampling on primary production and soil surface in North African rangelands. Ekológia Bratislava, 29, 219–226.

[ece38147-bib-0016] Ganskopp, D. , Cruz, R. , & Johnson, D. E. (2000). Least‐effort pathways?: A GIS analysis of livestock trails in rugged terrain. Applied Animal Behaviour Science, 68, 179–190. 10.1016/S0168-1591(00)00101-5 10804263

[ece38147-bib-0017] Gilad, E. , von Hardenberg, J. , Provenzale, A. , Shachak, M. , & Meron, E. (2004). Ecosystem engineers: From pattern formation to habitat creation. Physical Review Letters, 93, 098105‐1–098105‐4. 10.1103/PhysRevLett.93.098105 15447146

[ece38147-bib-0018] Halstead, B. J. , McCoy, E. D. , Stilson, T. A. , & Mushinsky, H. R. (2007). Alternative foraging tactics of juvenile gopher tortoises (*Gopherus* *polyphemus*) examined using correlated random walk models. Herpetologica, 63, 472–481.

[ece38147-bib-0019] Hoekstra, T. W. , & Shachak, M. (1999). Arid lands management: Toward ecological sustainability. University of Illinois Press.

[ece38147-bib-0020] Jin, B. , Sun, G. , Cheng, H. , Zhang, Y. , Zou, M. , Ni, X. , Luo, K. , Zhang, X. , Li, F. , & Wu, X. B. (2019). Goat track networks facilitate efficiency in movement and foraging. Landscape Ecology, 34, 2033–2044. 10.1007/s10980-019-00877-w

[ece38147-bib-0021] Jin, B. , Sun, G. , Zhang, Y. , Zou, M. , Ni, X. , Luo, K. , Zhang, X. , Cheng, H. , Li, F. , & Wu, B. X. (2016). Livestock tracks transform resource distribution on terracette landscapes of the Loess Plateau. Ecosphere, 7, e01337. 10.1002/ecs2.1337

[ece38147-bib-0022] Jones, C. G. , Lawton, J. H. , & Shachak, M. (1994). Organisms as ecosystem engineers. Oikos, 69, 373–386. 10.2307/3545850

[ece38147-bib-0023] Jones, C. G. , Lawton, J. H. , & Shachak, M. (1997). Positive and negative effects of organisms as physical ecosystem engineers. Ecology, 78, 1946–1957.

[ece38147-bib-0024] Kaligarič, M. , Brecl, J. , & Škornik, S. (2016). High potential of sub‐Mediterranean dry grasslands for sheep epizoochory. Open Life Sciences, 11, 177–184. 10.1515/biol-2016-0023

[ece38147-bib-0025] Lange, R. T. (1969). The piosphere: Sheep track and dung patterns. Journal of Range Management, 22, 396–400. 10.2307/3895849

[ece38147-bib-0026] Lickley, M. , & Solomon, S. (2018). Drivers, timing and some impacts of global aridity change. Environmental Research Letters, 13, 104010. 10.1088/1748-9326/aae013

[ece38147-bib-0027] Liu, J. , & Watanabe, T. (2013). Assessment of the current grazing intensity and slope status of pastures in the Alai Valley, Kyrgyzstan. Geographical Studies, 88, 70–79. 10.7886/hgs.88.70

[ece38147-bib-0028] Ludwig, J. A. , Tongway, D. J. , & Marsden, S. G. (1999). Stripes, strands or stipples: Modelling the influence of three landscape banding patterns on resource capture and productivity in semi‐arid woodlands, Australia. Catena, 37, 257–273. 10.1016/S0341-8162(98)00067-8

[ece38147-bib-0029] Meron, E. (2015). Nonlinear physics of ecosystems (344pp.). CRC Press.

[ece38147-bib-0030] Meron, E. (2016). Pattern formation – A missing link in the study of ecosystem response to environmental changes. Mathematical Biosciences, 271, 1–18. 10.1016/j.mbs.2015.10.015 26529391

[ece38147-bib-0031] Noy‐Meir, I. (1973). Desert ecosystems: Environment and producers. Annual Review of Ecology Evolution and Systematics, 4, 25–51. 10.1146/annurev.es.04.110173.000325

[ece38147-bib-0032] Pueyo, Y. , Moret‐Fernández, D. , Saiz, H. , Bueno, C. G. , & Alados, C. L. (2013). Relationships between plant spatial patterns, water infiltration capacity, and plant community composition in semi‐arid Mediterranean ecosystems along stress gradients. Ecosystems, 16, 452–466. 10.1007/s10021-012-9620-5

[ece38147-bib-0033] Rahmanian, S. , Hejda, M. , Ejtehadi, H. , Farzam, M. , Memariani, F. , & Pyšek, P. (2019). Effects of livestock grazing on soil, plant functional diversity, and ecological traits vary between regions with different climates in northeastern Iran. Ecology and Evolution, 9, 8225–8237. 10.1002/ece3.5396 31380085 PMC6662393

[ece38147-bib-0034] Ries, J. B. (2010). Methodologies for soil erosion and land degradation assessment in Mediterranean‐type ecosystems. Land Degradation & Development, 21, 171–187.

[ece38147-bib-0035] Ries, J. B. , Andres, K. , Wirtz, S. , Tumbrink, J. , Wilms, T. , Peter, K. D. , Burczyk, M. , Butzen, V. , & Seeger, M. (2013). Sheep and goat erosion – Experimental geomorphology as an approach for the quantification of underestimated processes. Zeitschrift Für Geomorphologie, 58, 23–45. 10.1127/0372-8854/2014/S-00158

[ece38147-bib-0036] Rosenthal, G. , Schrautzer, J. , & Eichberg, C. (2012). Low‐intensity grazing with domestic herbivores: A tool for maintaining and restoring plant diversity in temperate Europe. Tuexenia, 32, 167–205.

[ece38147-bib-0037] Schlesinger, W. H. , Reynolds, J. F. , Cunningham, G. L. , Huenneke, L. F. , Jarrell, W. M. , Virginia, R. A. , & Whitford, W. G. (1990). Biological feedbacks in global desertification. Science, 247, 1043–1048. 10.1126/science.247.4946.1043 17800060

[ece38147-bib-0038] Stavi, I. , Lavee, H. , Ungar, E. D. , & Sarah, P. (2009). Eco‐geomorphic feedbacks in semi‐arid rangelands: A review. Pedosphere, 19, 217–229. 10.1016/S1002-0160(09)60111-9

[ece38147-bib-0039] Stavi, I. , Lavee, H. , Ungar, E. D. , & Sarah, P. (2012). Grazing‐induced modification of a semi‐arid rangeland from a two‐phase to a three‐phase mosaic geo‐ecosystem. Arid Land Research and Management, 26, 79–83. 10.1080/15324982.2011.631691

[ece38147-bib-0040] Stavi, I. , Rachmilevitch, S. , Hjazin, A. , & Yizhaq, H. (2018). Geodiversity decreases shrub mortality and increases ecosystem tolerance to droughts and climate change. Earth Surface Processes and Landforms, 43, 2808–2817. 10.1002/esp.4412

[ece38147-bib-0041] Stavi, I. , Rachmilevitch, S. , & Yizhaq, H. (2018). Small‐scale geodiversity regulates functioning, connectivity, and productivity of shrubby, semi‐arid rangelands. Land Degradation & Development, 29, 205–209. 10.1002/ldr.2469

[ece38147-bib-0042] Stavi, I. , Shem‐Tov, R. , Chocron, M. , & Yizhaq, H. (2015). Geodiversity, self‐organization, and health of three‐phase semi‐arid rangeland ecosystems, in the Israeli Negev. Geomorphology, 234, 11–18. 10.1016/j.geomorph.2015.01.004

[ece38147-bib-0043] Stavi, I. , Ungar, E. D. , Lavee, H. , & Sarah, P. (2008). Grazing‐induced spatial variability of soil bulk density and content of moisture, organic carbon and calcium carbonate in a semi‐arid rangeland. Catena, 75, 288–296. 10.1016/j.catena.2008.07.007

[ece38147-bib-0044] Stavi, I. , Ungar, E. D. , Lavee, H. , & Sarah, P. (2009). Livestock modify ground surface microtopography and penetration resistance in a semi‐arid shrubland. Arid Land Research and Management, 23, 237–247. 10.1080/15324980903028371

[ece38147-bib-0045] Stavi, I. , Zinnes, T. A. , Joseph, A. , Solowey, E. , & Groner, E. (2015). The role of large herbivores in recruitment of Acacia trees via endozoochory in the Arava Valley, Israel. European Journal of Wildlife Research, 61, 775–781. 10.1007/s10344-015-0954-0

[ece38147-bib-0046] Sun, Y. , Hou, F. , Angerer, J. P. , & Yi, S. (2020). Effects of topography and land‐use patterns on the spatial heterogeneity of terracette landscapes in the Loess Plateau, China. Ecological Indicators, 109, 105839. 10.1016/j.ecolind.2019.105839

[ece38147-bib-0047] Tongway, D. J. , & Ludwig, J. A. (2003). The nature of landscape dysfunction in rangelands. In J. A. Ludwig , D. J. Tongway , D. Freudenberger , J. Noble , & K. Hodgkinson (Eds.), Landscape ecology function and management (pp. 49–61). CSIRO Publishing.

[ece38147-bib-0048] Trimble, S. W. , & Mendel, A. C. (1995). The cow as a geomorphic agent – A critical review. Geomorphology, 13, 233–253. 10.1016/0169-555X(95)00028-4

[ece38147-bib-0049] Turner, R. M. , Webb, R. H. , Bowers, J. E. , & Hastings, J. R. (2003). The changing mile revisited: An ecological study of vegetation change with time in the lower mile of an arid and semiarid region (p. 334). The University of Arizona Press.

[ece38147-bib-0050] Walker, J. W. , & Hkitschmidt, R. K. (1986). Effect of various grazing systems on type and density of cattle trails. Journal of Range Management, 39, 428–431. 10.2307/3899444

