# Supplementary material for: Positive impacts of livestock and wild ungulate routes on functioning of dryland ecosystems
Source: Ecol Evol. 2021 Sep 22;11(20):13684–91. doi: 10.1002/ece3.8147 (PMC8525128; doi:10.1002/ece3.8147)
Supplement: Supplementary file 1 — Appendix S1 [file ECE3-11-13684-s001.docx]

**Appendix A: Mathematical model describing the impact of trampling routes on runoff, soil-water, and vegetation biomass**

From a dynamic system point of view, landscape modification in the form of trampling routes can be regarded as a spatial periodic forcing problem, in which an external force with a given spatial period is imposed on a system that tends to develop its own natural period or wavelength. This is a classical resonance problem in physics (e.g., the periodically forced pendulum), which has been studied in the context of ecosystem rehabilitation (Mau et al., 2012; 2013; 2015). We modified the model that Mau et al. used to assess ecosystem rehabilitation by contour bench terraces (aka Shikim system: Meron, 2019; Meron et al., 2019) and the Australian fairy circles (Getzin et al., 2016). According to this model, the basic mechanism for vegetation pattern formation is the infiltration feedback known also as the overland flow mechanism, which is schematically illustrated in Figure A1. This mechanism is strengthened by the trampling routes, which are characterized by lower infiltration rate.


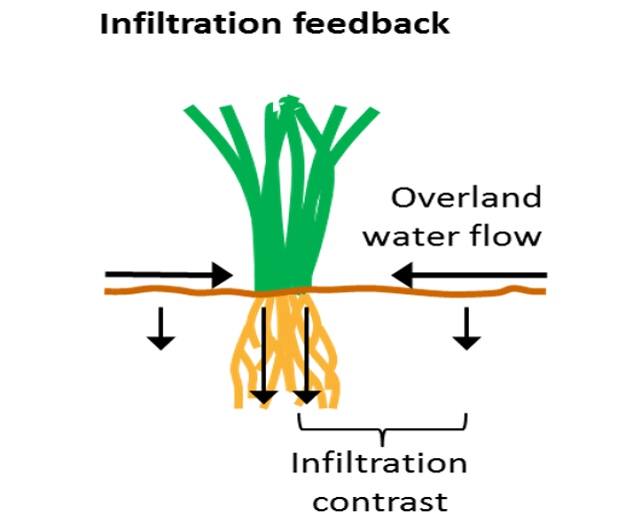


Fig. A1. Schematic illustration of the infiltration feedback. The bare soil area is covered by a physical or biological crust, and has lower infiltration. The overland water flows from the bare soil toward the vegetation patch. This sustains vegetation growth, but inhibits primary productivity in the inter-patch space.

The model can produce the basic periodic vegetation pattern along the precipitation gradient, as schematically shown in Figure A2. Bistability of different patterns under the same precipitation rate is predicted by the model, and also observed in the field (Fig. A3). To assess the effect of trampling routes, we studied the model under various precipitation rates using the stable pattern of banded vegetation on a moderate slope.


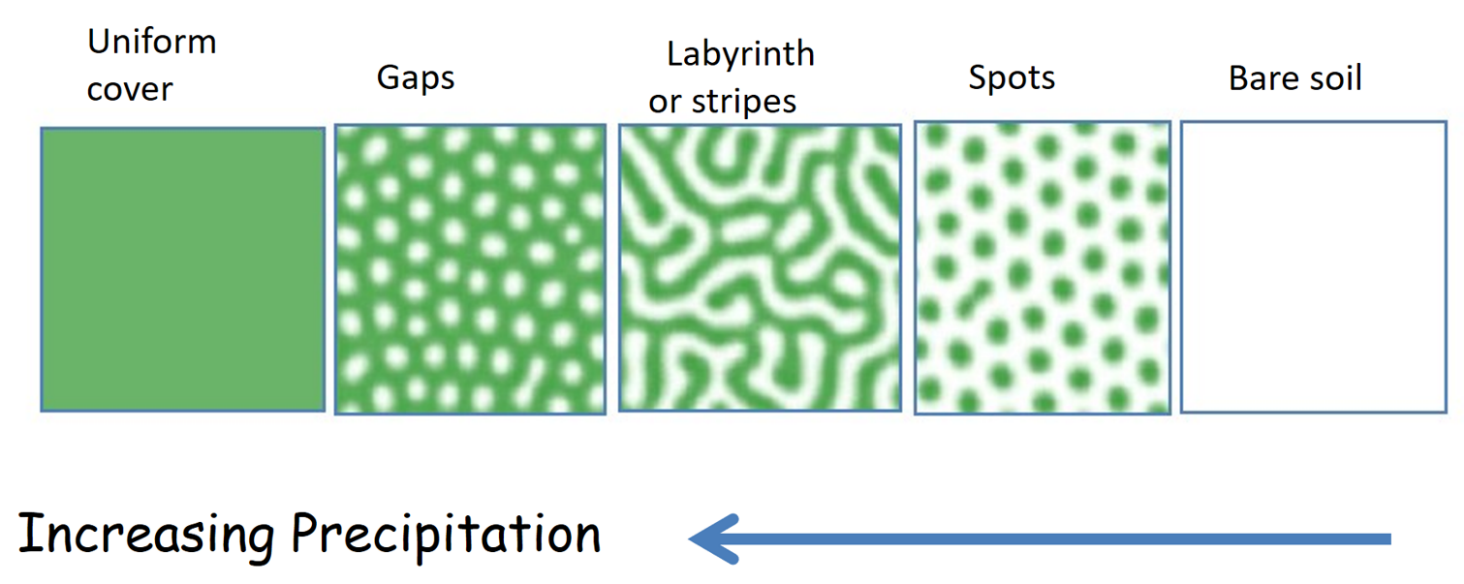


Fig. A2. The basic five self-organizing stable periodic vegetation patterns along the precipitation gradient that can be captured by the mathematical model. These patterns can emerge on completely homogenous substrate.


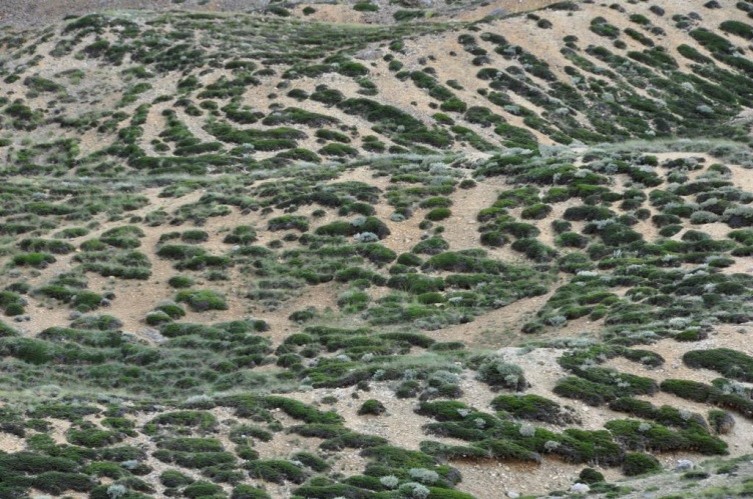


Fig. A3 Bistability of stripes and spots on a hillslope in Ladakh (India) at an altitude of 4000 m above sea level. The picture was taken by H. Yizhaq.

The model consists of a set of nonlinear partial differential equations that describe the coupled dynamics of three spatial densities: vegetation biomass (*B*), soil-water (*W*), and surface water (*H*), all in units of kg/m^2^. The model equations (modified from Gilad et al., 2007) are as follows (for more details, see Getzin et al. (2016) and Meron et al. (2019)).

$$\begin{matrix} \partial_{T}B=G_{B}B\left(1-\frac{b}{K}\right)-MB+D_{B}\nabla^{2}B \text{ }(1a) \\ \partial_{T}W=IH-L_{W}W-G_{W}W+D_{W}\nabla^{2}W \text{ }(1b) \\ \partial_{T}H=P-IH-L_{H}H+D_{H}\nabla^{2}H^{2}+2D_{H}\nabla H\cdot\nabla Z+2D_{H}H\nabla^{2}Z, (1c) \end{matrix}$$

where $T$ is time (years), $\nabla^{2}=\partial_{X}^{2}+\partial_{Y}^{2}$ is the Laplacian in the $(X,Y)$ plane, $\nabla=\partial_{X}\hat{X}+\partial_{Y}\hat{Y}$ is the gradient operator, is a partial derivative with respect to time, and $Z(X,Y)$is the is the ground topography function assumed to be independent on time (assuming no erosion or deposition processes). The derivation of the last two terms in Eq. (1c) is based on the shallow water equation. The shallow water approximation assumes a thin layer of water with negligible pressure variations so that the motion becomes almost two-dimensional (Weiyan, 1992). For flat topography, we remain with the term $D_{H}\nabla^{2}H^{2}$ (Eq. 1c), used for modeling Australian fairy circles and ring formation (Getzin et al., 2016; Yizhaq et al., 2019). The growth function $G_{B}$and the water uptake function $G_{W}$are given by:

$$G_{B}=\Lambda W(1+EB^{2}), G_{W}=\Gamma B(1+EB^{2})\text{ (2)}$$

The terms for evaporation from the soil ($L_{W}$), evaporation from the surface water ($L_{H}$), and soil-water infiltration ($I$) are given by:

$$L_{W}=\frac{N_{W}}{1+R_{W}B/K}, L_{W}=\frac{N_{H}}{1+R_{H}B/K}, I=A\frac{B+Qf}{B+Q}\text{ (3)}$$

The rest of the parameters in the model equations and the numerical values we used are presented in Table S1. To eliminate redundant parameters, we transformed the model equations (1a–3c) into a dimensionless form, by rescaling the variables and parameters. The dimensionless model equations read:

$$\begin{matrix} \partial_{t}b=g_{b}b(1-b)-b+\nabla^{2}b\text{ (3a)} \\ \partial_{t}w=Jh-l_{w}w-g_{w}w+\delta_{w}\nabla^{2}w\text{ (3b)} \\ \partial_{t}h=p-Jh-l_{h}h+\delta_{h}\nabla^{2}h^{2}\text{+}2\delta_{h}\nabla h\cdot\nabla z+2\delta_{h}h\nabla^{2}z\text{ , (3c)} \end{matrix}$$

where:

$l_{w}=\frac{\nu_{w}}{1+R_{w}b}, l_{h}=\frac{\nu_{h}}{1+R_{h}b}, J=\alpha\frac{b+qf}{b+q}, g_{b}=w(1+\eta b)^{2}, g_{w}=\gamma b(1+\eta b)^{2} (4)$.

Dimensionless quantities and the relations to their dimensional counterparts are shown in Table S2.

In order to model the trampling routes, we used a modified model for the parameter $f$that includes a periodically modulated infiltration rate (Meron et al., 2019). This modified parameter accounts for the decrease in infiltration over the trampling routes along the $x$ direction, i.e., perpendicular to the hillslope incline (Stavi et al., 2008, 2015), and can be calculated using Equation 5:

$f=f_{0}\left[1-\frac{\gamma_{f}}{2}\left(1+\cos(k_{f}x)\right)\right]$ (5)

where $f_{0}$ is the infiltration contrast of unmodulated soil, $\gamma_{f}$ is the modulation strength of the trampling routes, and $k_{f}$ is the wavenumber representing the spatial distribution of the trampling routes (their wavelength is given by $\lambda_{f}=2\pi k_{f}$).

According to this, the infiltration rate in densely vegetated areas is high, i.e., $I\approx A$, because the biomass density there is significantly higher than *Q*, a species-dependent reference value representing an >50% increase of the infiltration rate, whereas in bare soil ($B=0$) it is much lower, i.e., $I=Af_{0}$ in unmodulated bare soil, and $I=Af_{0}(1-\gamma{}_{f})$in bare soil with trampling routes. Thus, by this modeling approach, the system can be regarded as a three-phase mosaic, comprising of vegetation patch, interpatch spaces, and trampling route. Figure 4 shows the simulations for 5° hillslopes, with $\gamma=0.9$, and $f_{0}=0.01$, along a rainfall gradient in the system without trampling routes, where self-organized stripes developed from initially random conditions due to the Turing instability (a), and for systems with trampling routes, where the spatial forcing of the trampling routes forms straighter stripes (b). The models shows that average biomass density is higher in hillslopes with trampling routes, which act as an extra source of runoff water to the vegetation stripes. Thus, one of the important roles of trampling routes in drylands is to increase average biomass in the hillslopes, which can survive under lower precipitation rate.

Table S1. A list of model parameters used for the numerical simulations, their descriptions, units and typical numerical values (from Getzin et al., 2016)

| Parameter | Units | Description | Value |
| --- | --- | --- | --- |
| *K* | *kg/m^2^* | Maximum standing biomass | 0.666 |
| *Q* | *kg/m^2^* | Biomass reference value beyond which infiltration rate under a patch approaches its maximum | 1.2 |
| *M* | *yr^-1^* | Rate of biomass loss due to mortality and disturbances | 2 |
| *A* | *yr^-1^* | Infiltration rate in fully vegetated soil | 120 |
| *N_W_* | *yr^-1^* | Soil-water evaporation rate | 1.5 |
| *N_H_* | *yr^-1^* | Surface water evaporation | 4.5 |
| *E* | *m^2^/kg* | Root's augmentation per unit biomass | 1.5 |
| *Λ* | *m^2^/(kg$\cdot$yr)* | Biomass growth rate per unit soil-water | 0.03 |
| *Γ* | *m^2^/(kg$\cdot$yr)* | Soil-water use rate per unit biomass | 14 |
| *D_B_* | *m^2^/yr* | Seed dispersal coefficient | 0.02 |
| *D_W_* | *m^2^/yr* | Transport coefficient for soil-water | 2.5 |
| *D_H_* | *m^4^/(kg$\cdot$yr)* | Bottom friction coefficient between surface water and ground surface | 4 |
| *P* | *kg/(m^2^$\cdot$yr)* | Precipitation rate | variable |
| *R_W_* | *–* | Soil-water evaporation reduction due to shading | 0.3 |
| *R_H_* | *–* | Surface water evaporation reduction due to shading | 0.8 |
| *ƒ* | *–* | Infiltration contrast between bare soil and vegetated soil | 0.01 |

Table S2. Dimensionless quantities and the relations to their dimensional counterparts (from Getzin et al., 2016)

| Quantity | Scaling | Quantity | Scaling |
| --- | --- | --- | --- |
| $b$ | $B/K$ | $\gamma$ | $\Gamma K/M$ |
| $w$ | $W\Lambda/M$ | $p$ | $P\Lambda/M^{2}$ |
| $h$ | $H\Lambda/M$ | $\delta_{w}$ | $D_{W}/D_{B}$ |
| $q$ | $Q/K$ | $\delta_{h}$ | $D_{H}M/(D_{B}\Lambda)$ |
| $\nu_{w}$ | $N_{W}/M$ | $t$ | $TM$ |
| $\nu_{h}$ | $N_{H}/M$ | $x$ | $X\left( M/{D_{B}} \right)^{1/2}$ |
| $\alpha$ | $A/M$ | $y$ | $Y\left( M/{D_{B}} \right)^{1/2}$ |
| $\eta$ | $EK$ |  |  |
|  |  |  |  |

**References**

Getzin, S., Yizhaq, Y., Bell, B., Erickson, T.E., Postle, A.C., Katra, I., Tzuk, O., Zelnik, Y.R., Wiegand, K., Wiegand, T., Meron, E., 2016. Discovery of fairy circles in Australia supports self-organization theory. Proceedings of the National Academy of Sciences 113, 3551–3556.

Gilad E, von Hardenberg, J., Provenzale, A., Shachak, M., Meron, E., 2007. A mathematical model of plants as ecosystem engineers. Journal of Theoretical Biology 244, 680–691.

Mau, Y., Hagberg, A., Meron, E., 2012. Spatial periodic forcing can displace patterns it is intended to control. Physical Review Letters 109, 034102.

Mau, Y., Haim, L., Hagberg, A., Meron, E., 2013. Competing resonances in spatially forced pattern-forming systems. Physical Review E 88, 032917.

Mau, Y., Haim, L., Meron, E., 2015. Reversing desertification as a spatial resonance problem. Physical Review E 91, 012903.

Meron, E., Mau, Y., Zelnik, Y., 2019. Multistability in ecosystems: Concerns and opportunities for ecosystem function in variable environments. In: Kaper, H.G., Roberts, F.S., (eds.) Mathematics of Planet Earth 5.

Meron, E., 2019. Pattern-forming instabilities in dryland vegetation and their implications to ecosystem function and management. SIAM News, Volume 52, Number 7.

Stavi, I., Ungar, E.D., Lavee, H., Sarah, P., 2008. Grazing-induced spatial variability of soil bulk density and content of moisture, organic carbon and calcium carbonate in a semi-arid rangeland. Catena 75, 288-296.

Stavi, I., Shem-Tov, R., Chocron, M., Yizhaq, H., 2015. Geodiversity, self-organization, and health of three-phase semi-arid rangeland ecosystems, in the Israeli Negev. Geomorphology 234, 11-18.

Weiyan, T., 1992. Shallow Water Hydrodynamics. Elsevier Science, New York.

Yizhaq, H., Sela, S., Svoray T., Assouline, S., Bell, G., 2014. Effects of heterogeneous soil-water diffusivity on vegetation pattern formation. Water Resources Research 50, 5743–5758.

Yizhaq, H., Bel, G., 2016. Effects of quenched disorder on critical transitions in pattern-forming systems. New Journal of Physics 18, 023004.

Yizhaq, H., Stavi, I., Shachack, M., Bel, G., 2017. Geodiversity increases ecosystem durability to prolonged droughts. Ecological Complexity 31, 96–103.

Yizhaq, H., Stavi, I, Swet, N., Zaady, E., Katra, I. 2019. Overland water-flow mechanism for ring formation in water-limited environments: field measurements and mathematical modeling. Ecohydrology 12, e2135.
